# Testing-Yield Mismatch in Inpatient Micronutrient Assessment in Inflammatory Bowel Disease: A Real-World Implementation-Gap Analysis

**DOI:** 10.3390/nu18162648

**Published:** 2026-08-13

**Authors:** Amir Y. Kamel, Christopher Miquel-Chambers, Yasmeen Saker, Devika Dixit, Melanie Rolfe, Zachary D. Johnson, Isabela Hernandez, Thakul Rattanasuwan, Nofel Iftikhar, Naueen Chaudhry, Angela Pham, S. Devi Rampertab, Ellen Zimmermann

**Affiliations:** 1 Department of Pharmacy, University of Florida, Gainesville, FL 32610, USA; 2 Department of Internal Medicine, University of Florida, Gainesville, FL 32610, USA; 3 Department of Medicine, Division of Gastroenterology, Hepatology, and Nutrition, University of Florida, Gainesville, FL 32610, USA; 4 Department of Medicine, Miller School of Medicine, University of Miami, Miami, FL 33136, USA; 5 Department of Pharmacy Education and Practice, University of Florida College of Pharmacy, Gainesville, FL 32610, USA

**Keywords:** micronutrient, malnutrition, inflammatory bowel disease, vitamin deficiency, Crohn’s disease, ulcerative colitis, micronutrient, thiamine, pyridoxine, zinc, vitamin B12, implementation gap, quality improvement

## Abstract

**Background/Objectives**: Micronutrient deficiencies are common in inflammatory bowel disease (IBD) and contribute to anemia, impaired wound healing, neurologic injury, and adverse disease outcomes. Major societies, including ESPEN, ACG, and ECCO, recommend nutritional and micronutrient assessment in patients with IBD, yet few studies describe how comprehensively these recommendations are applied during hospitalization. This study aimed to characterize inpatient testing practices for vitamins B1, B6, B12, and zinc in hospitalized IBD patients, quantify deficiency frequency among those tested, and identify gaps between guideline-recommended and actual micronutrient assessment. **Methods**: A retrospective chart review was performed on adults with Crohn’s disease (CD) or ulcerative colitis (UC) hospitalized for an IBD flare at a tertiary care center. Demographics, comorbidities, and deficiency-associated clinical features were recorded. Yield was defined as the proportion of tested patients meeting institutional deficiency thresholds. Yield for each non-B12 micronutrient was compared with B12 as an internal benchmark. **Results**: Among 356 patients (285 CD, 71 UC), inpatient micronutrient testing was markedly imbalanced: B12 was tested in 97.2%, whereas B6, B1, and zinc were tested in only 34.8%, 30.6%, and 18.8%, respectively. Among those tested, deficiencies were common: B6 40.3%, zinc 32.8%, B1 22.9%, and B12 9.0%. Each non-B12 micronutrient yielded deficiency significantly more often per test than B12 (all *p* < 0.001), a testing-yield mismatch interpreted cautiously given selective testing of non-B12 nutrients. Deficiency frequencies did not differ significantly between CD and UC. Clinical contexts included tachycardia and edema (B1); elevated inflammatory markers and thromboembolism history (B6); anemia and neuropathy (B12); and diarrhea and hypoalbuminemia (zinc). **Conclusions**: Inpatient micronutrient assessment in IBD is heavily skewed toward B12, with B1, B6, and zinc under-tested despite a substantially higher yield of identified deficiency per test. This testing-yield mismatch represents a measurable implementation gap between guideline-recommended comprehensive assessment and actual inpatient practice; whether systematic panel-based assessment improves clinical outcomes warrants prospective evaluation.

## 1. Introduction

Inflammatory bowel disease (IBD) is strongly associated with malnutrition and micronutrient deficiencies, particularly during periods of active, severe disease [1,2,3]. The underlying mechanisms are multifactorial, including reduced oral intake, increased intestinal loss, malabsorption, hypermetabolic states driven by systemic inflammation, drug-nutrient interactions, and the consequences of prior bowel resection [1]. While macronutrient deficits are clinically important, micronutrient deficiencies are particularly prevalent and can persist even during remission and with seemingly adequate caloric intake [1]. Deficiencies of B vitamins and zinc have been linked to anemia, peripheral and central neuropathy, thrombophilia, impaired wound healing, osteoporosis and colorectal cancer [1], and malnutrition in hospitalized IBD patients has been associated with prolonged length of stay and higher mortality [2].

Reflecting this clinical importance, major gastroenterology and clinical nutrition societies recommend nutritional evaluation in IBD, though the specificity and scope of recommendations vary. The 2023 European Society for Clinical Nutrition and Metabolism (ESPEN) guideline recommends that IBD patients be screened for micronutrient deficiencies at least annually, using both clinical and laboratory evaluation, with specific attention to iron, zinc, and vitamin D [4]. The American College of Gastroenterology (ACG) clinical guidelines for IBD recommend baseline laboratory evaluation that includes assessment of anemia, inflammation, and malnutrition [5,6]. The European Crohn’s and Colitis Organisation (ECCO) consensus on anemia and micronutrient deficiency specifies annual measurement of B12 and folate with more frequent testing for high-risk phenotypes such as small-bowel disease or prior resection [7]. Additionally, the ECCO extraintestinal manifestations guideline reinforces attention to non-iron micronutrients [8]. The 2024 American Gastroenterological Association (AGA) Clinical Practice Update on Diet and Nutritional Therapies recommends monitoring vitamin D, iron, and B12 (in patients with ileal disease or resection), while identifying zinc, copper, and fat-soluble vitamins as important to consider depending on individual risk factors—a tiered approach that underscores the need for data on which micronutrients are actually being assessed in practice [9].

Despite these recommendations, few studies have described how comprehensively micronutrient assessment is carried out during IBD hospitalization, a period when patients are most ill, least orally tolerant, and most vulnerable to nutrient depletion. A recent AGA Clinical Practice Update on inpatient IBD management further highlights nutritional risk as a key component of supportive inpatient care [10]. Hospitalization for flare represents both an opportunity to correct ongoing nutritional deficits and a vulnerable window in which missed deficiencies can contribute to preventable morbidity. From a clinical standpoint, the most consequential question in IBD nutrition is whether correcting specific deficiencies alters disease trajectory. Vitamin D provides the clearest example: supplementation has been linked to fewer IBD-related emergency visits, hospitalizations, and courses of corticosteroids, and meta-analysis supports a reduction in clinical relapse [11,12]. Comparable interventional evidence remains limited for B1, B6, B12, and zinc. A necessary precursor to generating it is documenting whether these nutrients are measured at all during the highest-risk window of inpatient flare care. The aim of this study was to characterize real-world inpatient testing practices for vitamins B1, B6, B12, and zinc in adults hospitalized for an IBD flare at a tertiary care center, quantify the frequency of identified deficiencies among those tested, describe the clinical contexts in which deficiencies were recognized, and identify specific gaps between guideline-recommended comprehensive assessment and actual inpatient practice that may be addressable through quality-improvement interventions.

## 2. Materials and Methods

A single-center retrospective electronic chart review was performed on adults with an ICD-9 or ICD-10 diagnosis code for Crohn’s disease (CD) or ulcerative colitis (UC) who were hospitalized for an IBD flare at a tertiary care center between 1 January 2013 and 30 June 2017.

### 2.1. Inclusion and Exclusion Criteria

Patients were included if they:

1. Were admitted with symptoms consistent with an IBD flare;
2. Had an ICD-9/10 code for a diagnosis of CD or UC during their index hospitalization;
3. Had ≥1 micronutrient level (serum or whole blood) measured during the hospitalization.

Patients were excluded if they:

1. Were pregnant or incarcerated;
2. Had micronutrient testing performed for an unrelated medical condition;
3. Had incomplete medical records or an uncertain IBD diagnosis.

### 2.2. Data Collection

Patients’ age, gender, height, total body weight (TBW), ideal body weight (IBW), body mass index (BMI), IBD type, intestinal disease location, and history of fistula, stricture, or surgery were collected. Whole blood thiamine (B1) and serum pyridoxine (B6) levels were measured with high-performance liquid chromatography–tandem mass spectrometry, whereas serum zinc levels were measured using inductively coupled plasma-mass spectrometry (ARUP Laboratories, Salt Lake City, UT, USA). Serum vitamin B12 levels were measured by competitive-binding immunoassay on the Access/DxI platform (Beckman Coulter, Inc., Brea, CA, USA) in the UF Health clinical laboratory. Micronutrient laboratory testing was performed selectively based on clinical suspicion at the discretion of the treating medical team, reflecting real-world physician-triggered testing practices. Standard institutional reference ranges were applied to define deficiency.

### 2.3. Outcomes

The primary outcome was the yield of each micronutrient test—defined as the proportion of tested patients whose result met the institutional threshold for deficiency—together with the proportion of the cohort tested for each micronutrient. The secondary outcome was the characterization of clinical features documented in patients with identified deficiency. Because testing was physician-triggered rather than protocolized, yield is reported as a property of the test as it was ordered and is not interpreted as population prevalence.

### 2.4. Statistical Analysis

Descriptive statistics were applied using JMP version 16.0 (SAS Institute Inc., Cary, NC, USA). Means and standard deviations were calculated for continuous variables, while categorical variables were described using frequencies and percentages.

Throughout this manuscript, testing rate denotes the proportion of the full cohort in whom a given micronutrient was measured, and yield denotes the proportion of tested patients whose result met the institutional threshold for deficiency. Yield is a within-cohort measure conditional on having been tested and is distinct from population prevalence. Within-nutrient deficiency yield (proportion of tested patients identified as deficient) was summarized with 95% Wilson score confidence intervals. Pre-specified pairwise comparisons of yield for each non-B12 micronutrient versus B12, and an overall four-group yield comparison, were performed using Pearson’s chi-square tests. Two-sided Fisher’s exact tests were used instead for comparisons where cell counts were sparse, namely the CD versus UC deficiency comparisons. Yield comparisons were interpreted cautiously, as B12 was tested in nearly all patients while the non-B12 nutrients were tested selectively on the basis of clinical suspicion; selection bias likely inflates yield estimates for the selectively tested nutrients relative to B12, and the comparisons should therefore be understood as quantifying the observed testing-yield mismatch in routine practice rather than as unbiased population-level deficiency rates. Multivariable logistic regression was considered but not performed, as structured patient-level data for key confounders—including C-reactive protein, serum albumin trajectory, cumulative corticosteroid exposure, prior bowel resection as a modeled variable, concurrent Clostridioides difficile and cytomegalovirus infection, and dietary intake—were not available in the retrospective dataset. Performing regression using only the available variables would omit these clinically important confounders and risk producing biased or overinterpreted adjusted estimates. Accordingly, all inferential analyses are unadjusted and intended to be hypothesis-generating. The study is therefore positioned as an implementation-gap analysis quantifying real-world testing behavior and within-cohort yield disparities, not as a prevalence estimate; this framing is reflected throughout the Results and Discussion.

### 2.5. Ethical Considerations

This study was approved by the University of Florida Institutional Review Board on 31 August 2017 (IRB# 201701678; PI: Dr. Amir Kamel) with waiver of informed consent due to its retrospective design.

## 3. Results

**Figure 1:**
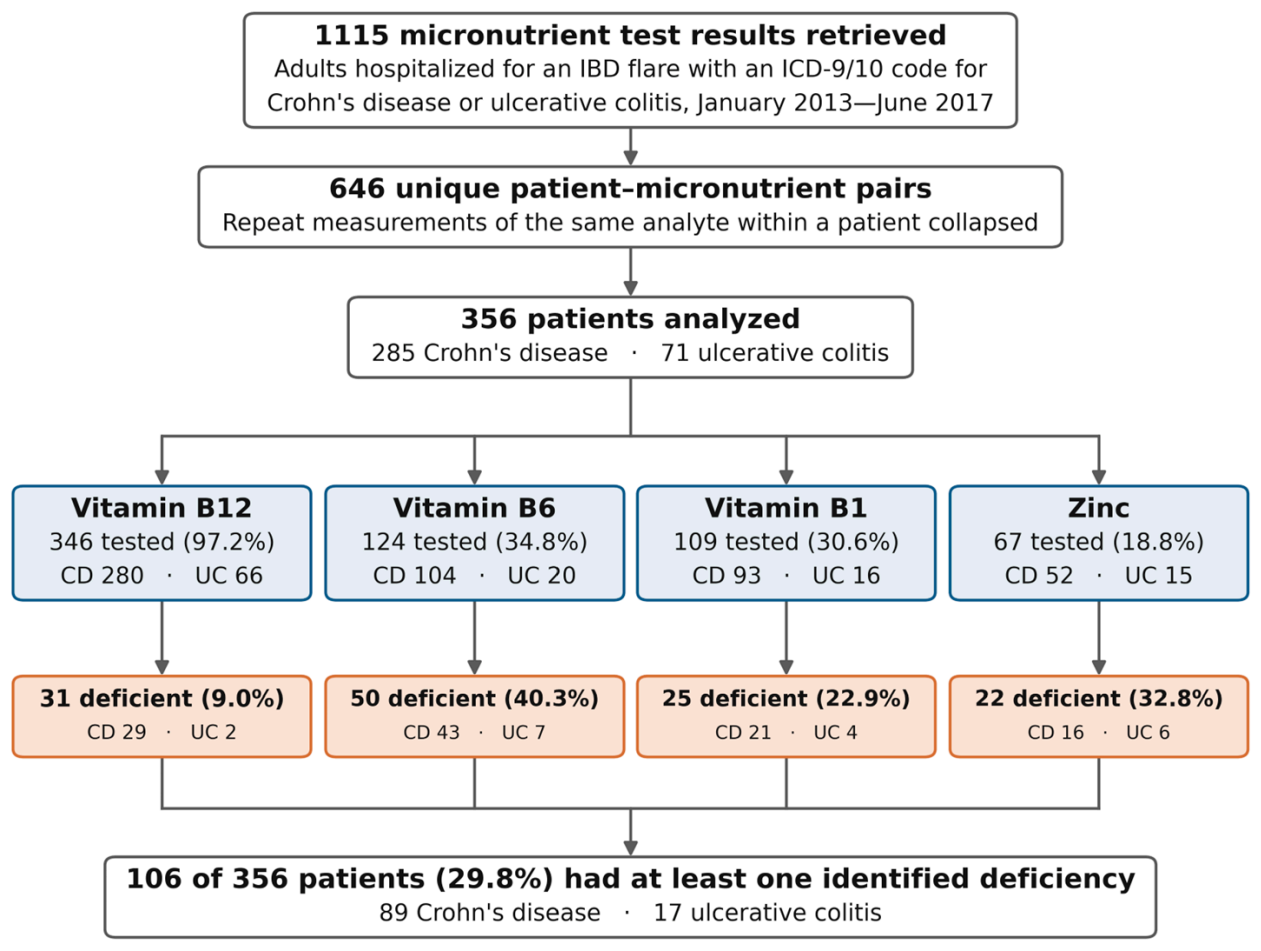
Study flow chart. Micronutrient test results retrieved, unique patient–micronutrient pairs, patients analyzed, and patients with identified deficiency for each micronutrient, by IBD subtype. Testing rates are expressed as a proportion of the 356 analyzed patients, and deficiency counts as a proportion of those tested for that micronutrient.

**Figure 2:**
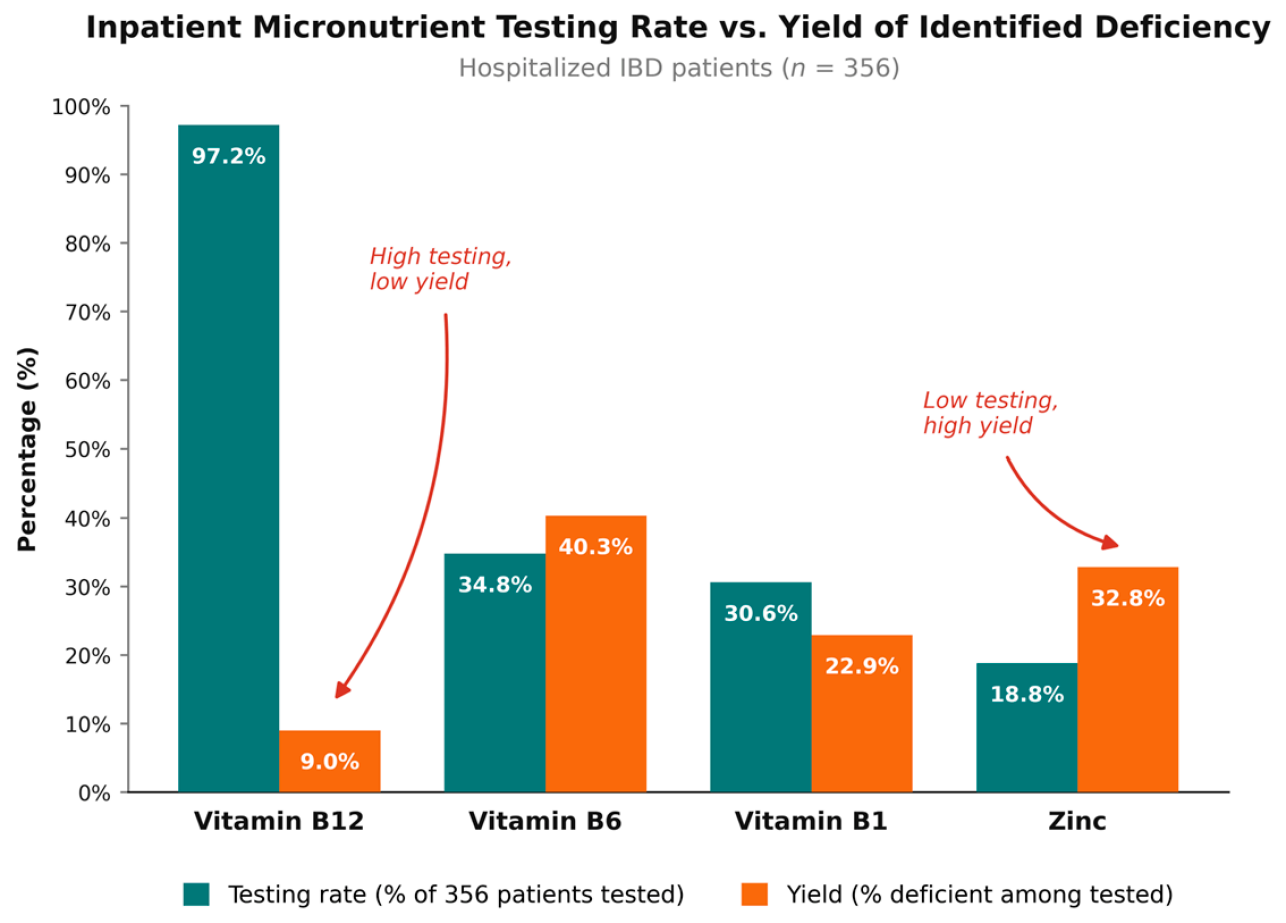
Inpatient micronutrient testing rate versus yield of identified deficiency among hospitalized IBD patients (*n* = 356). B12 was tested near-universally but yielded deficiency in 9.0% of tests, whereas B6, B1, and zinc were tested in a minority of patients and yielded deficiency in 40.3%, 22.9%, and 32.8% respectively (each vs. B12, chi-square *p* < 0.001).

During the study period, 1115 micronutrient test results were retrieved for hospitalized patients with an IBD diagnosis code. These corresponded to 646 unique patient-micronutrient pairs in 356 individual patients (285 CD, 71 UC), all of whom had at least one micronutrient level obtained during the index admission. Overall, 106 (29.8%) had at least one micronutrient deficiency with a mean age of 45 years. (Figure 1) Among patients with deficiency, 64 were female and 42 were male. Demographic characteristics of the study population are listed in Table 1. Micronutrient testing frequency varied: vitamin B12 was tested in 346 patients (97.2%), vitamin B6 in 124 patients (34.8%), vitamin B1 in 109 patients (30.6%), and zinc in 67 patients (18.8%). A total of 21 of 93 CD patients (22.6%) and 4 of 16 UC patients (25%) had thiamine deficiency (*p* = 0.759). A total of 43 of 104 CD patients (41.3%) and 7 of 20 UC patients (35%) had pyridoxine deficiency (*p* = 0.630). Vitamin B12 was found to be deficient in 10.4% of CD and 3% of UC patients (*p* = 0.09), whereas zinc was found to be deficient in 30.8% of CD patients and 40% of UC patients respectively (*p* = 0.542) (Table 2a,b). Testing patterns showed a marked imbalance across micronutrients: B12 was measured in nearly all patients, while B1, B6, and zinc were checked in fewer than half of the hospitalized cohort, yet identified deficiencies were common when these tests were obtained (yield when tested with 95% Wilson CI: B12 9.0%, 6.4–12.4%; B6 40.3%, 32.1–49.1%; zinc 32.8%, 22.8–44.7%; B1 22.9%, 16.0–31.7%). In pre-specified pairwise yield comparisons versus B12, each non-B12 micronutrient yielded significantly more deficiency per test than B12 (B6 vs. B12: odds ratio [OR] 6.87, chi-square *p* < 0.001; zinc vs. B12: OR 4.97, *p* < 0.001; B1 vs. B12: OR 3.02, *p* < 0.001), with a significant overall four-group difference (chi-square *p* < 0.001). These comparisons should be interpreted as quantifying the observed testing-yield mismatch in real-world practice, since clinicians preferentially order non-B12 tests when deficiency is suspected, which likely inflates yield estimates for selectively tested nutrients relative to B12. The testing rate versus yield of identified deficiency for each micronutrient is summarized in Table 3 and illustrated in Figure 2, which display the underlying counts, Wilson CIs, and pairwise *p*-values. The clinical features most commonly documented in patients with identified deficiencies, stratified by the IBD type, are presented in Table 4. The most commonly documented clinical features in patients with identified deficiency were diarrhea and infection (zinc); depression (B6, B1); history of venous thromboembolism (B6); tachycardia and edema (B1); and anemia and peripheral neuropathy (B12).

## 4. Discussion

In this real-world single-center retrospective cohort of 356 hospitalized adults with IBD, we observed a substantial imbalance in inpatient micronutrient assessment. Vitamin B12 was measured in nearly all patients (97.2%), whereas thiamine (B1), pyridoxine (B6), and zinc were each measured in fewer than half (30.6%, 34.8%, and 18.8%, respectively). When obtained, deficiencies were common, particularly for B6, (41.3% in CD, 35.0% in UC) and zinc (30.8% in CD, 40.0% in UC). Notably, the yield of identified deficiency per test performed was substantially and significantly higher for the under-tested micronutrients than for B12, with B6 yielding deficiency nearly seven-fold more often per test (OR 6.87, *p* < 0.001), zinc almost five-fold (OR 4.97, *p* < 0.001), and B1 three-fold (OR 3.02, *p* < 0.001). We acknowledge that clinicians preferentially test B1, B6, and zinc when deficiency is clinically suspected, which likely enriches yield estimates for those nutrients relative to B12 (which is tested near-universally); this selection effect does not fully explain the observed gap, as even a markedly attenuated true yield difference would leave these nutrients substantially under-recognized in routine inpatient practice. This pattern suggests that routine inpatient care disproportionately focuses on a single micronutrient while under-assessing others with established clinical relevance in IBD.

This study adds to a limited body of literature quantifying real-world micronutrient testing patterns during IBD hospitalization. Prior work from our group quantified testing rates and deficiency incidence for copper and vitamins A, B9, E, and K in this cohort, demonstrating that these micronutrients are not routinely assessed during IBD hospitalization [13]. The present analysis extends those findings to B1, B6, and zinc and is methodologically distinct in two respects: (i) it introduces B12—the only near-universally tested micronutrient in our cohort—as an internal benchmark against which the testing yield of selectively assessed nutrients can be compared within the same patient population, and (ii) it explicitly frames the observed testing imbalance as a quality of care implementation gap rather than as a prevalence estimate.

### 4.1. A Gap Between Guideline Recommendations and Real-World Inpatient Practice

Major gastrointestinal and nutrition societies endorse nutritional evaluation in IBD, though the scope and specificity of recommendations differ. The 2023 European Society for Clinical Nutrition and Metabolism (ESPEN) guidelines on clinical nutrition in IBD recommends that patients be checked for micronutrient deficiencies regularly, including in remission, with both clinical and laboratory evaluation, and identifies iron, zinc, and vitamin D as deficiencies that commonly require specific replacement [4]. The American College of Gastroenterology (ACG) clinical guideline on Crohn’s disease similarly specifies that initial laboratory evaluation should include assessment for inflammation, anemia, dehydration, and malnutrition [5], and the ACG ulcerative colitis guideline highlights baseline laboratory assessment and nutritional status evaluation during disease assessment [6]. The European Crohn’s and Colitis Organisation (ECCO) consensus on iron deficiency and anemia in IBD recommends measurement of B12 and folate at least annually, with closer surveillance for those with small-bowel disease or resection [7], while the ECCO extraintestinal manifestations guideline emphasizes that micronutrient deficiencies contribute to a broad range of systemic complications beyond anemia alone [8]. The 2024 AGA Clinical Practice Update takes a tiered approach, recommending routine monitoring of vitamin D, iron, and B12 (in ileal disease or resection) while identifying zinc and other micronutrients as important to consider based on individual risk factors [9]. The observed testing pattern in our cohort—with near-universal B12 testing but infrequent zinc, B1, and B6 testing—may therefore partly reflect guideline-concordant prioritization of B12 in at-risk phenotypes, but the very low testing rates for zinc (18.8%) and B1 (30.6%) fall short even of the risk-stratified assessment these guidelines envision. The divergence between guideline recommendations for comprehensive assessment and observed inpatient behavior represents a tractable target for quality improvement, particularly given that hospitalization for flare is a time when patients are most ill, least orally tolerant, and most vulnerable to micronutrient depletion.

### 4.2. The Malnutrition–Inflammation–Immunity Triad and Its Surgical Corollary

Reliance on B12 alone as a proxy for overall micronutrient status is biologically unsupported. B1, B6, B12, and zinc occupy overlapping but non-redundant roles in a malnutrition-inflammation-immunity triad—a framework reflecting the interconnected pathways through which reduced intake, systemic inflammation, and immune dysregulation simultaneously drive and are exacerbated by micronutrient depletion in IBD. Anorexia, diarrhea, protein-losing enteropathy, and mucosal inflammation simultaneously reduce intake and increase losses of water-soluble vitamins and trace elements. Concurrent enteric infection during IBD flare—most notably Clostridioides difficile and cytomegalovirus—further amplifies losses, particularly of zinc and thiamine, and is itself associated with worse short-term outcomes; while these co-infections were not systematically captured in our retrospective dataset, their contribution to acute micronutrient depletion is well-recognized and reinforces rather than diminishes the case for inpatient assessment. Systemic inflammation in turn lowers circulating levels of several nutrients (including B6 and zinc) independent of dietary intake [14,15]; and depletion of any one of these micronutrients can in turn impair mucosal barrier function, innate and adaptive immune responses, and wound healing [1,16,17]. Zinc deficiency in IBD has been independently associated with subsequent hospitalization, surgery, and disease-related complications, with normalization associated with improved outcomes [18]; low B6 in IBD has been linked to thrombotic risk and active disease [15]; and thiamine depletion during catabolic stress carries a risk of irreversible neurologic injury [19]. Because the shared drivers of deficiency (reduced intake, malabsorption, inflammation, increased losses) act on multiple nutrients simultaneously, a normal B12 does not exclude depletion of B1, B6, or zinc. This biological interdependence argues for a panel-based rather than single-analyte approach during flare-related hospitalization. This framework has a direct surgical corollary: a recent systematic review of post-operative infectious complications in IBD identified preoperative malnutrition, hypoalbuminemia, and corticosteroid exposure as among the most consistent risk factors for post-operative infection [20], underscoring that the malnutrition-inflammation axis operative during hospitalized flare extends directly into perioperative outcome modification. To the extent that hospitalized flare frequently precedes urgent or semi-urgent surgery, inpatient micronutrient recognition is plausibly relevant not only to in-hospital recovery but to downstream surgical risk.

### 4.3. Clinical Implications

Our findings support several practical changes in inpatient IBD care. First, B12 testing alone should not be considered an adequate nutritional assessment during IBD flare; guideline-concordant evaluation also requires consideration of B1, B6, zinc, iron, and vitamin D [4,5,6,7,8]. Second, institutions should consider whether admission order sets and flare-management pathways embed structured micronutrient assessment rather than relying on clinical recall, which our data suggest is inconsistent. Third, because B6 and zinc behave as negative acute-phase reactants, hospitalized values should be interpreted alongside concurrent inflammatory markers, and repeat measurement after inflammation resolves is needed to distinguish transient suppression from persistent depletion [4]. The AGA Clinical Practice Update encourages checking inflammation-sensitive micronutrients during quiescent disease [9], which creates a practical tension: the flare patient is simultaneously the most likely to have low circulating levels and the most likely to benefit from recognition and replacement of true deficiency. Rather than deferring all testing to remission, a reasonable approach is to flag low values during hospitalization, initiate replacement when clinically indicated, and confirm persistent deficiency once inflammation resolves. Fourth, collaboration with clinical nutrition services—already recommended by ESPEN—may help standardize assessment and follow-up across admissions and outpatient transitions [4].

Empiric supplementation may be worth considering for selected micronutrients whose serum levels are difficult to interpret during active inflammation. Because B6 and zinc behave as negative acute-phase reactants, measured levels during a flare may not accurately reflect tissue stores, and waiting for quiescent disease to test may delay recognition and treatment of true deficiency. Prior work from our group has suggested that prophylactic oral zinc and pyridoxine supplementation may be considered in IBD patients susceptible to deficiencies during flares, with monitoring for secondary copper deficiency [21]. A recent randomized controlled trial demonstrated that daily supplementation with B1, B6, B12, and magnesium reduced fatigue and UC disease activity scores compared to placebo, though notably in patients with quiescent rather than active disease [22]. However, current guidelines recommend test-and-treat rather than empiric replacement [4,9], and excess zinc supplementation during flare carries a risk of copper deficiency. Whether a hybrid strategy—empiric supplementation during flare with confirmatory testing during remission—improves outcomes while maintaining safety warrants prospective evaluation.

### 4.4. Thiamine (Vitamin B1)

Thiamine is absorbed in the jejunum and ileum and has limited body stores (2–3 weeks), making hospitalized IBD patients vulnerable to rapid depletion during periods of poor oral intake or increased metabolic demand [23]. Deficiency is well-described in chronic alcoholism and prolonged parenteral nutrition but less frequently considered in IBD, despite case reports of Wernicke encephalopathy in malnourished CD patients receiving unsupplemented parenteral nutrition [19] and evidence that 32% of CD patients in remission have diminished thiamine levels [24]. An open-label pilot study and a subsequent randomized controlled trial both demonstrated benefit of high-dose oral thiamine for chronic fatigue in quiescent IBD [25,26]. In our cohort, approximately one quarter of tested patients were thiamine-deficient, with tachycardia, edema, and peripheral neuropathy the most commonly documented features—findings that overlap substantially with flare physiology and may be easily missed without directed laboratory evaluation. Given the irreversibility of neurologic sequelae, a low threshold for measurement in hospitalized IBD patients appears warranted, particularly with poor oral intake, vomiting, or diuretic use.

### 4.5. Pyridoxine (Vitamin B6)

Pyridoxal 5′-phosphate (PLP), the active form of vitamin B6, serves as a cofactor for over 140 biochemical reactions and is absorbed throughout the small intestine [27]. Circulating PLP behaves as a negative acute-phase reactant, falling with systemic inflammation independent of dietary intake [15]. B6 was the most frequently identified deficiency among tested patients in our cohort (41.3% of CD, 35.0% of UC), most often documented in patients with elevated inflammatory markers and, less commonly, depressive symptoms or prior venous thromboembolism—consistent with prior reports linking low PLP to inflammation and thrombotic risk in IBD [14,15]. Low plasma B6 during a flare likely reflects a combination of true tissue depletion and inflammation-driven redistribution; this does not argue for deferring testing but rather for recognizing that the hospitalized flare patient is more likely to be B6-depleted and for confirming status once acute inflammation resolves. Prior studies have reported B6 deficiency in 14–29% of IBD patients, with dietary inadequacy accounting for only a minority of cases, suggesting that malabsorption and inflammation are the primary drivers [28,29].

### 4.6. Cobalamin (Vitamin B12)

Vitamin B12 is absorbed in the distal ileum and has large body stores (3–5 years), so serum levels during a flare largely reflect longer-term status rather than acute inflammation [30,31]. Patients with ileal CD or ileal resection are at particular risk, and both ECCO and ESPEN recommend at least annual monitoring with more frequent assessment in high-risk phenotypes [4,7]. In our cohort, B12 was the most frequently tested nutrient yet yielded the lowest rate of identified deficiency (9.0%), with low B12 most commonly documented in patients with anemia, peripheral neuropathy, or paresthesia. Because B12 is (a) near-universally tested, (b) not acutely suppressed by inflammation, and (c) stored in large quantities, it serves as a clean benchmark against which the inadequate inpatient testing of B1, B6, and zinc can be measured. Prior studies report B12 deficiency prevalence of 15.6% in CD versus 2.8% in UC, with ileal resection as a key risk factor [30,32]. Oral cyanocobalamin is effective replacement therapy in CD [33].

### 4.7. Zinc

Zinc is absorbed primarily in the duodenum and jejunum and plays essential roles in immune function, wound healing, and mucosal barrier integrity [1,18]. Zinc loss in IBD is driven by high-output diarrhea, protein-losing enteropathy, and mucosal inflammation, and ESPEN specifically identifies zinc as a deficiency requiring targeted replacement [4]. Importantly, zinc deficiency has been independently associated with subsequent hospitalization, surgery, and disease-related complications in a prospectively followed IBD cohort, with outcomes improving after normalization [18]. Like B6, serum zinc behaves as a negative acute-phase reactant, falling rapidly through IL-6-driven hepatic sequestration [4]. In our cohort, zinc was tested in the smallest proportion of patients (18.8%) yet identified deficiency in nearly one third of those tested, most often in the presence of high-volume diarrhea, hypoalbuminemia, or prior bowel resection—a proportion broadly consistent with zinc deficiency prevalence reported in other Crohn’s disease cohorts [34]. The combination of high clinical consequence, low testing rates, and high yield when tested makes zinc a particularly compelling target for structured inpatient assessment. Because all inferential analyses were unadjusted and limited to tested patients, the clinical features described in Section 4.4, Section 4.5, Section 4.6 and Section 4.7 should be interpreted as contexts in which deficiency was identified, rather than as independent predictors of deficiency.

### 4.8. Limitations

This study has several important limitations that should be considered when interpreting these findings. By design, this is a single-center retrospective cohort with physician-triggered rather than protocolized testing; the reported deficiency frequencies therefore apply only to tested patients and should not be interpreted as population-level prevalence in all hospitalized IBD patients. This selective testing also likely enriches for deficiency among tested individuals, since clinicians preferentially order levels when deficiency is clinically suspected. Second, because virtually all patients in this cohort were admitted for an IBD flare, systemic inflammation was essentially ubiquitous, and the four micronutrients studied differ in their susceptibility to acute-phase confounding. Serum zinc and plasma pyridoxal 5′-phosphate (B6) behave as negative acute-phase reactants: zinc falls within hours through IL-6-driven hepatic sequestration and metallothionein upregulation, and PLP falls through tissue redistribution and reduced albumin binding, both largely independent of body stores [4,7,18,28]. Thiamine body stores are limited (2–3 weeks) so low values during critical illness more plausibly reflect true depletion, though compartmental shifts have been described. Vitamin B12, in contrast, is not acutely suppressed by inflammation and has large body stores (3–5 years’ worth), so B12 levels in our cohort are unlikely to be artifactually lowered by flare physiology. Observed low B6 and zinc values in our cohort therefore likely reflect a combination of true tissue depletion and inflammation-driven redistribution that cannot be disentangled in retrospective data. Critically, however, acute-phase suppression does not argue for withholding testing during a flare: the flare patient is both the most likely to have low circulating levels and the most likely to benefit from recognition and replacement of any underlying true deficiency, and repeat measurement once inflammation resolves can distinguish transient from persistent deficits. Third, we did not include a remission or non-IBD control group, which precludes inference about whether the identified deficiencies are specific to acute flares or reflect broader patterns of chronic illness and inflammation. This design choice reflects the primary objective, which was to characterize real-world inpatient testing practices, not to estimate disease-specific prevalence. Fourth, structured data on inflammatory markers (CRP), albumin trajectories, corticosteroid exposure, prior bowel resection coded for regression, diet, Clostridioides difficile and cytomegalovirus co-infection, and nutrition consult utilization were not available for multivariable analysis; we therefore did not perform regression modeling, as unadjusted associations in a selectively tested cohort would be prone to bias and overinterpretation. Fifth, because multiple micronutrients were often tested concurrently in each patient, we cannot fully disentangle the contribution of individual versus combined deficiencies to observed symptom patterns. Sixth, symptom documentation relied on retrospective abstraction from clinical notes, which may under-report milder manifestations. Seventh, we did not capture whether identified deficiencies led to supplementation or other clinical action, which would be relevant to the quality-improvement framing of these findings. In addition, detailed disease phenotyping (Montreal classification) and standardized disease-severity scoring at admission were not uniformly documented in the retrospective record; this precludes reliable phenotype and severity-stratified analysis and prospective standard phenotyping with validated activity indices is a priority for future multicenter work. Finally, single-center data may not generalize to practice patterns at other institutions; however, the testing imbalance we describe is concordant with clinical experience and with the limited published data on inpatient micronutrient assessment, motivating multicenter evaluation.

## 5. Conclusions

In this real-world cohort of hospitalized adults with IBD, vitamin B12 was tested in nearly all patients while thiamine (B1), pyridoxine (B6), and zinc were measured in fewer than half, despite deficiencies being common when obtained. This testing imbalance represents a gap between current gastroenterology and clinical nutrition guideline recommendations for comprehensive micronutrient evaluation and actual inpatient practice. Because B1, B6, B12, and zinc occupy interconnected roles in what we describe as the malnutrition-inflammation-immunity triad, a normal B12 does not exclude other clinically important deficiencies, and panel-based rather than single-analyte assessment is biologically justified. Embedding structured micronutrient evaluation into inpatient IBD care pathways, potentially through order-set redesign and routine clinical nutrition collaboration, is a tractable quality-improvement opportunity. Whether systematic inpatient assessment of this micronutrient panel translates into improved short-term recovery, reduced postoperative infectious complications, or reduced disease-related rehospitalization warrants prospective, multicenter evaluation. Our data should be regarded as defining the implementation gap that such trials will need to close, rather than as evidence of disease-specific prevalence.

## Figures and Tables

**Table 1:** Patient demographics.

	All Patients	Micronutrient Deficient Patients	CD			UC		
	*n* = 356	*n* = 106	All CD Patients	Non-Deficient	Deficient	All UC Patients	Non-Deficient	Deficient
Characteristic			*n* = 285	*n* = 196	*n* = 89	*n* = 71	*n* = 54	*n* = 17
Age, y, mean (range)	48 (18–92)	45 (19–79)	47 (19–92)	48 (19–92)	44 (19–79)	52 (18–84)	52 (18–84)	53 (24–79)
Female, *n* (%)	187 (52.5)	64 (60.4)	152 (53.3)	92 (47.0)	60 (67.4)	35 (49.3)	31 (57.4)	4 (23.5)
Weight, kg, mean ± SD	73.5 ± 21.9	71.3 ± 23.2	73.9 ± 22.6	75.9 ± 21.9	70.4 ± 23.6	72.1 ± 19.0	70.5 ± 18.2	76.1 ± 21.1
Body mass index, kg/m^2^; mean ± SD	26.0 ± 7.39	24.9 ± 7.61	26.15 ± 7.68	26.91 ± 7.53	24.86 ± 7.81	25.36 ± 6.10	25.36 ± 5.94	25.31 ± 6.67
Length of stay, mean days (range)	10.0 (0–96)	8.6 (0–56)	10.6 (0–85)	11.0 (1–85)	9.5 (0–56)	8.2 (2–96)	9.3 (2–96)	5.3 (2–15)
Alcohol use, *n* (%)								
Yes	71 (19.9)	22 (20.8)	66 (23.2)	45 (23.0)	21 (23.6)	5 (7.0)	4 (7.4)	1 (5.9)
No	212 (59.6)	58 (54.7)	162 (56.8)	117 (59.7)	45 (50.6)	50 (70.4)	37 (68.5)	13 (76.5)
Not asked	73 (20.5)	26 (24.5)	57 (20.0)	34 (17.3)	23 (25.8)	16 (22.5)	13 (24.1)	3 (17.6)
Tobacco use, *n* (%)								
Yes/passive	50 (14.0)	14 (13.2)	44 (15.4)	32 (16.3)	12 (13.5)	6 (8.5)	4 (7.4)	2 (11.8)
Quit	93 (26.1)	24 (22.6)	70 (24.6)	51 (26.0)	19 (21.3)	23 (32.4)	18 (33.3)	5 (29.4)
Never	146 (41.0)	44 (41.5)	119 (41.8)	82 (41.8)	37 (41.6)	27 (38.0)	20 (37.0)	7 (41.2)
Not asked	67 (18.8)	24 (22.6)	52 (18.2)	31 (15.8)	21 (23.6)	15 (21.1)	12 (22.2)	3 (17.6)

Values are mean (range) or *n* (%) unless otherwise noted. CD, Crohn’s disease; UC, ulcerative colitis.

**Table 2:** (**a**) Frequency of identified micronutrient deficiency among tested IBD patients. (**b**) Micronutrient test volume, unique patients tested, and unique patients with identified deficiency, by IBD subtype.

(**a**)							
**Deficiency Frequency (%)**							
		**CD**		**UC**		**CD vs. UC *p*-Value**	
B1 (*n* = 109)		22.6		25.0		0.76	
B6 (*n* = 124)		41.3		35.0		0.63	
B12 (*n* = 346)		10.4		3.0		0.09	
Zinc (*n* = 67)		30.8		40.0		0.54	
(**b**)							
		**Zinc**	**B1**	**B6**	**B12**	**Total**	**Total After Removing Duplicates Across Micronutrients**
Total entries							
	CD	78	113	152	587	930	
	UC	19	23	24	119	185	
	Subtotal	97	136	176	706	1115	
Total unique entries							
	CD	52	93	104	280	529	285
	UC	15	16	20	66	117	71
	Subtotal	67	109	124	346	646	356
Unique deficient							
	CD	16	21	43	29	109	89
	UC	6	4	7	2	19	17
	Subtotal	22	25	50	31	128	106

IBD, inflammatory bowel disease; CD, Crohn’s disease; UC, ulcerative colitis.

**Table 3:** Inpatient micronutrient testing rate versus yield of identified deficiency among tested patients.

Micronutrient	Patients Tested, *n* (%)	Deficient Among Tested, *n* (%)	Yield % (95% CI)	OR (*p*-Value) vs. B12
Vitamin B12	346 (97.2)	31 (9.0)	9.0 (6.4–12.4)	referent
Vitamin B6	124 (34.8)	50 (40.3)	40.3 (32.1–49.1)	6.87 (*p* < 0.001)
Vitamin B1	109 (30.6)	25 (22.9)	22.9 (16.0–31.7)	3.02 (*p* < 0.001)
Zinc	67 (18.8)	22 (32.8)	32.8 (22.8–44.7)	4.97 (*p* < 0.001)

Testing rate = patients tested/356 total hospitalized IBD patients. Yield when tested = deficient patients/tested patients. 95% confidence intervals (CIs) for yield are Wilson score intervals. Odds ratios (OR) and *p*-values compare yield of each non-B12 micronutrient versus B12 using Pearson’s chi-square test, with B12 as the referent. B12 was tested in the majority of patients but yielded deficiency in a small minority, while B1, B6, and zinc were tested in a minority of patients but yield deficiency substantially more often per test. Because testing for B1, B6, and zinc was physician-driven and typically prompted by clinical suspicion, yield estimates for these nutrients likely reflect enriched populations and should not be generalized to all hospitalized IBD patients; the magnitude of the yield disparity nonetheless illustrates a testing-yield mismatch relevant to inpatient quality of care.

**Table 4:** Clinical features documented in patients with identified micronutrient deficiency.

**Zinc**			**B6**		
	**CD**	**UC**		**CD**	**UC**
**Total**	**16**	**6**	**Total**	**43**	**7**
Zinc value, reference range (mcg/dL)	60–130		B6 value, reference range (nmol/L)	20–125	
Zinc value of deficient patients, mean ± SD (mcg/dL)	49.2 ± 7.8	47.4 ± 6.5	B6 value of deficient patients, mean ± SD (nmol/L)	10.6 ± 5.0	9.2 ± 5.9
Zinc value of non-deficient patients, mean ± SD (mcg/dL)	81.6 ± 17.2	72.1 ± 10.7	B6 value of non-deficient patients, mean ± SD (nmol/L)	46.2 ± 53.4	56.0 ± 41.7
Taste/Smell Alterations	3 (19)	0 (0)	Increased Inflammatory Markers (CRP, TNF-α, IL-6)	29 (67)	5 (71)
Skin Lesions	2 (13)	1 (17)	Rheumatoid Arthritis	1 (2)	0 (0)
Bowel Resection	6 (38)	3 (50)	Glossitis	1 (2)	1 (14)
Diarrhea	12 (75)	4 (67)	Cheilitis	2 (5)	2 (29)
Diarrhea > 5× per day	5 (31)	1 (17)	Somnolence (Presented with)	2 (5)	1 (14)
Rash	7 (44)	0 (0)	Depression (Presented with)	10 (23)	1 (14)
Hair Loss	3 (19)	0 (0)	Peripheral Neuropathy	3 (7)	1 (14)
Fistulas	4 (25)	0 (0)	MI (history of)	0 (0)	1 (14)
Albumin Deficient (<3.6 g/dL)	8 (50)	5 (83)	Ischemic Stroke (history of)	4 (9)	1 (14)
In ICU During Admission	2 (13)	2 (33)	DVT/PE (history of)	9 (21)	3 (43)
Infection	4 (25)	2 (33)	Steroid use	22 (51)	0 (0)
Sepsis	1 (6)	1 (17)			
**B1**			**B12**		
	**CD**	**UC**		**CD**	**UC**
**Total**	**21**	**4**	**Total**	**29**	**2**
B1 value, reference range (nmol/L)	70–180		B12 value, reference range (pg/mL)	243–894	
B1 value of deficient patients, mean ± SD (nmol/L)	26.0 ± 16.2	24.3 ± 13.1	B12 value of deficient patients, mean ± SD (pg/mL)	196.7 ± 51.1	217 ± 35.4
B1 value of non-deficient patients, mean ± SD (nmol/L)	135.7 ± 55.8	114.3 ± 40.6	B12 value of non-deficient patients, mean ± SD (pg/mL)	719.3 ± 464.7	835.5 ± 492.4
Peripheral neuropathy	5 (24)	3 (75)	Anemia	26 (90)	1 (50)
Cardiomyopathy (Beri Beri)	1 (5)	1 (25)	Megaloblastic anemia	1 (3)	0 (0)
Calf muscle tenderness	3 (14)	1 (25)	Peripheral neuropathy	16 (55)	1 (50)
Edema	6 (29)	1 (25)	Paresthesia	13 (45)	2 (100)
Tachycardia	13 (62)	2 (50)	Gait disturbances	3 (10)	0 (0)
Cardiomegaly	2 (10)	0 (0)	Cognitive Impairment	6 (21)	1 (50)
HF	3 (14)	1 (25)	Depression	12 (41)	0 (0)
CHF	2 (10)	1 (25)	Myalgias	9 (31)	0 (0)
Represented as *n* (%) unless otherwise noted					

Values are *n* (%) unless otherwise noted. Features that were sought and not documented in any patient are not listed: alcohol use exceeding 14 drinks per week (vitamin B6); and pancytopenia, ataxia, cerebral manifestations, altered mental status, psychosis, paraplegia, and dementia (vitamin B12). CD, Crohn’s disease; CHF, congestive heart failure; CRP, C-reactive protein; DVT, deep vein thrombosis; HF, heart failure; IL-6, interleukin-6; MI, myocardial infarction; PE, pulmonary embolism; TNF-α, tumor necrosis factor alpha; UC, ulcerative colitis.

## Data Availability

Data supporting the findings of this study are available from the corresponding author upon reasonable request and in accordance with institutional policies.
